# NMR chemical shift assignment of the IMLV methyl groups of a di-domain of the Tomaymycin non-ribosomal peptide synthetase

**DOI:** 10.1007/s12104-025-10231-8

**Published:** 2025-04-25

**Authors:** John P. Kirkpatrick, Megha N. Karanth, Teresa Carlomagno

**Affiliations:** 1 School of Biosciences, College of Life and Environmental Sciences, University of Birmingham, Birmingham, B15 2TT UK; 2 Department of Cancer and Genomic Sciences, School of Medical Sciences, College of Medicine and Health, University of Birmingham, Birmingham, B15 2TT UK; 3 Institute of Organic Chemistry and Centre of Biomolecular Drug Research, Leibniz University of Hannover, Schneiderberg 38, 30167 Hannover, Germany; 4 Helmholtz Institute for Infection Research, Inhoffenstraße 7, 38124 Braunschweig, Germany

**Keywords:** Tomaymycin, Non-ribosomal peptide synthetases, Methyl assignment, Condensation domain

## Abstract

Non-ribosomal peptide synthetases (NRPSs) are macromolecular enzymatic complexes responsible for the biosynthesis of an array of microbial natural products, many of which have important applications for human health. The nature of the NRPS machinery, which has been likened to an assembly line, should be amenable to bio-engineering efforts directed towards efficient synthesis of novel and tailored molecules. However, the success of such endeavours depends on a detailed understanding of the mechanistic principles governing the various steps in the peptide assembly-line. Here, we report the near-complete assignment of the Ile, Met, Leu and Val methyl-groups of the 59-kDa adaptor-condensation di-domain (BN-BC) from the Tomaymycin NRPS. These assignments will provide the foundation for future NMR studies of the complex dynamic behaviour of the condensation domain both in isolation and in the context of the enzymatic cycle, which will themselves form the basis for developing a complete mechanistic description of the central condensation reaction in this prototypical NRPS.

## Biological context

Non-ribosomal peptides are a class of natural products produced by bacteria and fungi that exhibit a wide range of pharmacological activity (Süssmuth and Mainz 2017) and have been extensively utilized for medicinal use, for example as anti-bacterial and anti-cancer agents. The biosynthesis of these peptides occurs in multiple steps via large protein assemblies known as non-ribosomal peptide synthetases (NRPSs).

**Fig. 1:**
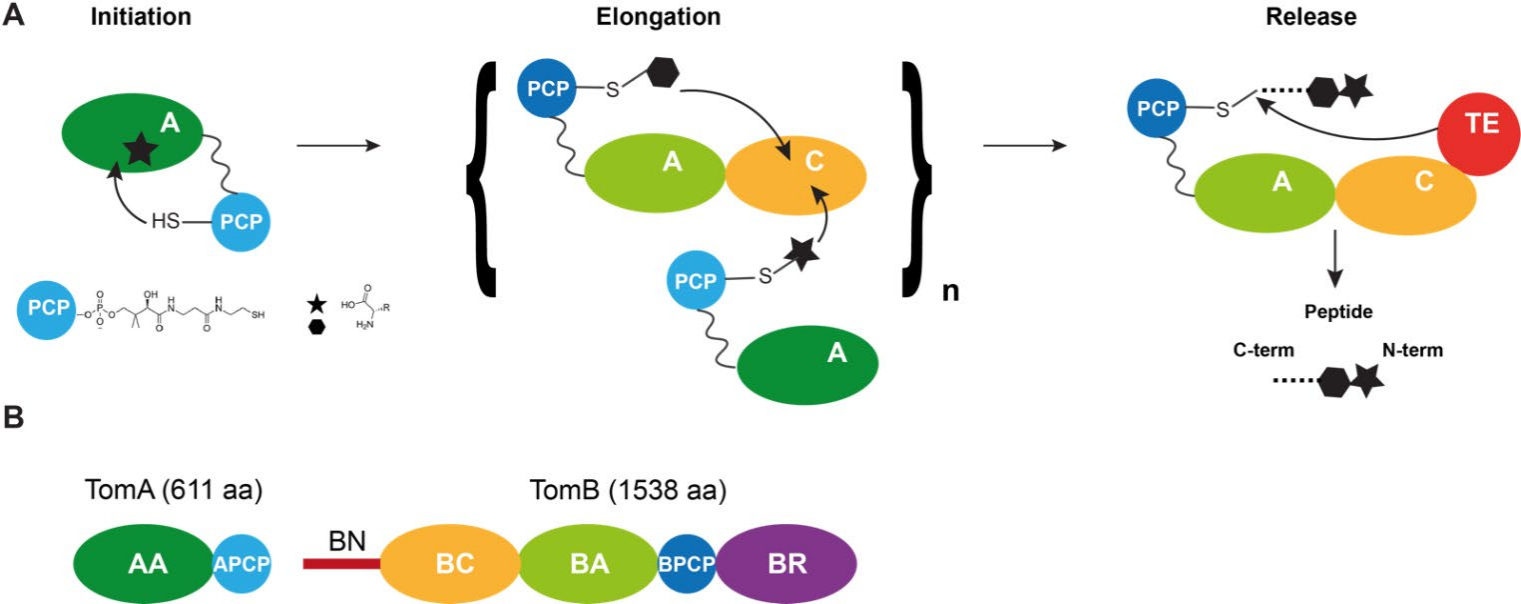
**(A)** Schematics of the assembly-line-like process leading to the synthesis of non-ribosomal peptides. During Initiation, the first non-canonical amino acid is loaded on the first PCP by the adenylation domain of the first module. The following modules contain at least one PCP, one adenylation and one condensation domain each. They may also contain tailoring domains that modify the amino acid added by the module. The adenylation domain of each module loads the corresponding PCP with the non-canonical amino acid specific to that module and the condensation domain links the amino acid loaded on the PCP of the preceding module (donor PCP) to that on the PCP of the same module (acceptor PCP). The acceptor PCP carries the elongated chain and interacts with the next module in the role of donor PCP. This process can happen several times. The last module contains a thioesterase domain that releases the peptide from the last PCP. **(B)** Domain architecture of the Tomaymycin NRPS, which consists of two modules, TomA and TomB (AA/BA: adenylation domain of TomA/B; APCP/BPCP: PCP domain of TomA/B; BC: condensation domain of TomB; BN: N-terminal domain of TomB; BR: reductase domain of TomB). The thioesterase domain is substituted by a reductase domain that releases the product

NRPS enzymes are composed of modules, wherein one module is responsible for a particular amino-acid building block. Modules are further divided into discrete domains having a specific function, these being: an ATP-dependent adenylation domain (A) that has substrate/amino-acid specificity (Fig. 1A); a condensation domain (C) that catalyses peptide-bond formation (Fig. 1A); a thioesterase domain (TE) that catalyses the release of the final peptide (Fig. 1A); tailoring domains that can carry out specific modifications; and finally a peptidyl-carrier-protein (PCP) domain. The PCP domain uses its 4-phosphopantetheine prosthetic group (Fig. 1A) to shuttle either the nascent peptide chain or the next amino-acid to be added to the different reaction centres both within and across modules. The synthesis then proceeds via precise coordination of both domains and modules within time and space.

This modular nature of non-ribosomal peptide synthesis makes NRPS enzymes amenable to protein reengineering to synthesize designer drug molecules, and also to find sustainable and non-toxic routes for synthesis of common chemicals. A fundamental hindrance to such efforts is the incomplete understanding of the structural and dynamic behaviour of the NRPS domains, and in particular the rules governing their mutual recognition and interactions.

In this work, we have studied the condensation domain of the two-module NRPS (called TomA-B) that produces the anticancer drug Tomaymycin (Arima et al. 1972; von Tesmar et al. 2017; Karanth et al. 2024) (Fig. 1B). Condensation domains, which are approximately 50 kDa in size, mediate intermodular communication by binding to two different PCP domains — the donor PCP from the upstream module and the acceptor PCP from its own module — and then catalysing the peptide-bond formation between the two payloads (Bloudoff and Schmeing 2017). The condensation domain has two similar-sized sub-domains (the N- and C-lobes), creating a central tunnel that houses the active-site residues. The two ends of this tunnel provide entry points for the substrate-laden donor and acceptor PCPs. Available structures of condensation domains indicate that while these domains show minimal sequence similarity across different NRPSs, the overall structural scaffold is preserved (Keating et al. 2002; Bloudoff et al. 2013). There remain however important aspects of structural variability, such as differences in the relative orientation of the N- and C-lobes, as well as in the arrangements of the loops that guard the entry points at either end of the catalytic tunnel and that hence modulate the accessibility of the active site (Reimer et al. 2019; Patel et al. 2023). The large size and dynamic nature of these condensation domains poses significant challenges to their study by solution-state NMR, there being only one other study on a structurally similar but functionally different cyclization domain to date (Mishra et al. 2022). In the TomA-B system, the condensation domain belongs to the TomB module and is therefore called BC (Fig. 1B). We have recently reported the discovery of a novel adaptor domain on the N-terminal side of the BC domain (called BN) that is essential for the specific recruitment of the upstream PCP domain of the TomA module to the condensation domain (Karanth et al. 2024) (Fig. 1B). We report here the ILVM methyl-group assignments of the di-domain construct BN-BC, providing the basis for future NMR-based investigations into the structural and dynamic properties of this important NRPS.

## Methods and experiments

### Sample Preparation

#### Constructs and labelling schemes

The BN, BC and BN-BC constructs correspond to residues 1–90, residues 91–533 and residues 1–533 of the TomB module. With the exception of the sample used for the HmCmCG/CB experiment, all BN-BC and BC samples were selectively “methyl-labelled”, i.e. uniformly deuterated and ^12^C-labelled at all positions except those corresponding to a subset of methyl groups, which were selectively protonated and ^13^C-labelled according to one of the following labelling schemes (non-^1^H/^13^C-labelled methyl-groups were ^2^H/^12^C-labelled): Ile-δ1 alone (“I-only”), racemic Leu-δ1/δ2 & Val-γ1/γ2 (“LV[rac]”); Ile-δ1, racemic Leu-δ1/δ2 & racemic Val-γ1/γ2 (“ILV[rac]”); Ile-δ1, Met-ε, racemic Leu-δ1/δ2 & racemic Val-γ1/γ2 (“IMLV[rac]”); Met-ε & stereospecific Val-γ2 (“MV[pro-S]”); stereospecific Leu-δ1 & Val-γ1 (“LV[pro-R]”). The sample for the HmCmCG/CB experiment was uniformly deuterated and ^12^C-labelled for all residues except for Leu and Val, which were uniformly deuterated and methyl-labelled in a similar manner to the LV[rac] scheme described above, but whose non-methyl side-chain carbons (with the exception of Leu-C′ & Leu-C^α^) were ^13^C-labelled; this labelling scheme is called “LV[rac;^13^C-sc]” in what follows. All BN-BC Ile point-mutant samples were prepared with I-only labelling, while all BN-BC Leu and Val point-mutant samples were prepared with LV[rac] labelling. The labelling schemes for the other BN-BC and BC samples are given together with the experiments for which they were used in the section “Pulse sequences and acquisition parameters” below. BN was prepared with uniform ^13^C- and ^15^N-labelling.

#### Protein expression and purification

All proteins were produced by over-expression in *E. coli* BL21(DE3) cells from pETM44 vectors as N-terminal hexa-histidine tagged MBP fusion proteins with an HRV3C protease cleavage site between MBP and the protein of interest, as described previously (Karanth et al. 2024). Briefly, cultures were initially grown at 37°C with shaking at 180 rpm to an OD_600_ of 0.6–0.8. Uniformly ^15^N,^13^C-labelled BN was prepared using 1 g/L of ^15^N ammonium chloride and 3 g/L of ^13^C D-glucose in the M9 minimal medium. Cultures for methyl-labelled samples and the sample with LV[rac;^13^C-sc] labelling were grown with 3 g/L of ^2^H glycerol in 99% D_2_O. All cultures were then cooled to 16°C and protein expression was induced by addition of 0.1 mM IPTG (isopropyl β-D-1-thiogalactopyrranoside); the cells were incubated for a further 18–20 hrs at 16°C. To produce the methyl- and LV[rac;^13^C-sc]-labelled samples, appropriate precursors were added to the cultures one hour prior to induction (Goto et al. 1999): racemic Leu-δ1/δ2 & Val-γ1/γ2 labelling was obtained by addition of 120 mg/L of [3-^13^C; 3,4,4,4,-D_4_]-2-ketoisovalerate (Cambridge Isotope Laboratories); Ile-δ1 labelling was obtained by addition of 60 mg/L of [3-^13^C;3,3-D_2_]-2-ketobutyrate (Cambridge Isotope Laboratories); LV[rac;^13^C-sc] labelling was obtained by addition of 120 mg/L of [1,2,3,4-^13^C_4_; 3,4’,4’,4’,-D_4_]-2-ketoisovalerate (Cambridge Isotope Laboratories); Met-ε labelling was obtained by addition of [2,3,3,4,4,-D_5_,^13^C] methyl-L-methionine (NMRBio); stereospecific Leu-δ1 & Val-γ1 labelling was obtained by addition of 2-[D_3_]methyl-2,4-^13^C_2_-acetolactate (NMRBio); stereospecific Val-γ2 labelling was obtained by addition of [D_10_]-leucine and 2-[^13^C]-methyl-4-[D_3_]-acetolactate (NMRBio). NMRBio precursors were added in the quantities recommended by the manufacturer. Cells were harvested by centrifugation, resuspended in lysis buffer (50 mM Tris–HCl (tris(hydroxymethyl)aminomethane–HCl) pH 8.0, 1 M NaCl, 1 mM DTT (dithiothreitol), 5% v/v glycerol), supplemented with 1x EDTA-free protease-inhibitor-cocktail tablet (Roche), and with DNAase I (Merck) and lysozyme (Merck) to 10 µg/ml and 100 µg/ml of resuspended pellet, respectively. Cells were then lysed by high-pressure homogenization and the clarified lysates purified by passing through a 5-ml HisTrap HP column (Cytiva Life Sciences) equilibrated in wash buffer (50 mM Tris–HCl pH 8.0, 1 M NaCl,) using an ÄKTA Pure protein purification system (Cytiva Life Sciences). Bound protein was eluted with a linear 0–300mM gradient of imidazole in wash buffer, dialyzed against wash buffer and incubated with His-HRV-3 C protease (1:100 protease: protein w/w ratio) overnight. The protease and cleaved MBP tag were removed by affinity chromatography (5-ml HisTrap HP column), with the protein of interest collected from the flow-through, concentrated and further purified by size-exclusion chromatography (HiLoad Superdex 200 pg 16/60 or Superdex 200 Increase 10/300 GL columns, Cytiva Life Sciences) using H_2_O- or D_2_O-based NMR buffer (50 mM sodium phosphate pH 7.0, 150 mM NaCl, 0.5–1 mM TCEP (tris(2-carboxyethyl)phosphine), 0.02% w/v NaN_3_). Peak fractions at each purification step were checked for purity by SDS PAGE (sodium dodecyl sulfate polyacrylamide gel electrophoresis).

Point mutations in BN-BC were generated by site-directed mutagenesis of the wild-type construct using protocols described in QuikChange II kit from Agilent Technologies and confirmed by DNA sequencing. The following single-point mutants were generated for the purpose of providing anchor-point assignments: I53A, I80A, V95A, I104A, I107A, L159A, L183A, V187A, I221A, L256A, L270A, L328A, L340A, V334A, V363A, L365A, L381A, I388A, V394A, L409A, I449A, I485A, V497A, and L504A. The following double-point mutants were generated for measurement of BN-BC intra-protein paramagnetic relaxation enhancements (PREs): [C32S]S102C, [C32S]A185C and [C32S]S406C (C32 was identified as surface-exposed using the program NACCESS (Hubbard and Thornton n.d.) and was therefore mutated to a serine in these three PRE constructs.

#### NMR samples

Final protein concentrations varied according to the experiments to be recorded and were in the range 50–800 µM. Samples were loaded into one of four different types of NMR tubes as required (approximate sample volumes in parentheses): 3-mm diameter standard tubes (180 µL), 5-mm diameter standard tubes (540 µL), 3-mm diameter Shigemi tubes (110 µL) and 5-mm diameter Shigemi tubes (330 µL).

Paramagnetic samples for PRE measurements were made by conjugating the terminal sulfhydryl group of the target cysteine residue to the nitroxide-based spin-label 3-(2-iodoacetamido)-PROXYL (3-(2-Iodoacetamido)-2,2,5,5-tetramethyl-1-pyrrolidinyloxy, free radical) (Sigma-Aldrich). Briefly, pure protein samples were completely reduced by incubation with a 100-fold molar excess of DTT for several hours. The DTT was then removed by rapid buffer exchange (using a desalting column) into 50 mM Tris-HCl pH 8.0, 150 mM NaCl, followed by immediate addition of a 10-fold molar excess of 3-(2-iodoacetamido)-PROXYL. The sample was then incubated overnight in the dark at 16 °C, after which unreacted spin-label was removed by buffer exchange into NMR buffer and the sample concentrated as required. After recording a ^1^H,^13^C-HMQC spectrum on the sample in its paramagnetic state, the nitroxide radical of spin-label was reduced by direct addition of ascorbic acid to a final concentration of 10 mM, after which the ^1^H,^13^C-HMQC spectrum was recorded (with the same acquisition parameters) on the sample in its resulting diamagnetic state.

### Data acquisition

#### Spectrometers

NMR spectra were acquired on Bruker NMR spectrometers operating at ^1^H static field-strengths of 600 MHz (Avance III HD console), 850 MHz (Avance III HD console) and 1000 MHz (Avance Neo console), equipped with nitrogen-cooled (600-MHz spectrometer) or helium-cooled (850-MHz & 1000-MHz spectrometers) cryogenic inverse 5-mm HCN probeheads and running Bruker Topspin v3.2 (600-MHz & 850-MHz spectrometers) or v4.1 (1000-MHz spectrometer). Most of the spectra were recorded on the 850-MHz spectrometer; the ^1^H,^13^C-HMQC spectra of point-mutants I80A & I221A were recorded on the 600-MHz spectrometer and those of point-mutants V95A, L256A and L340A were recorded on the 1000-MHz spectrometer.

#### Pulse sequences and acquisition parameters

All 2D ^1^H,^13^C-methyl spectra of BN-BC (and BC) were recorded using a ^1^H,^13^C-HMQC pulse sequence (Mueller 1979; Robin Bendall et al. 1983; Tugarinov et al. 2003); the highest-resolution methyl spectra were recorded with a modified version of the pulse sequence that incorporated a purge element to actively remove contributions to the final spectrum arising from coherence-transfer pathways involving fast-relaxing coherences (Korzhnev et al. 2004), which otherwise manifest themselves as line-broadening at the base of the peaks. Typical acquisition times (*t*_1,max_) for 2D ^1^H,^13^C-methyl spectra were 80–100 ms and 40–60 ms in the ^1^H and ^13^C dimensions, respectively. The 2D ^1^H,^13^C spectrum of uniformly ^13^C,^15^N-labelled BN was recorded using a constant-time ^1^H,^13^C-HSQC pulse sequence (Santoro and King 1992; Vuister and Bax 1992).

3D HCH- and CCH-NOESY spectra were recorded on an IMLV[rac]-labelled BN-BC sample (~ 800 µM in a 5-mm Shigemi tube) with uniform sampling in all dimensions. The HCH-NOESY spectrum was recorded using a ^13^C-HMQC–NOESY pulse sequence (Fesik and Zuiderweg 1988; Ikura et al. 1990) that incorporated double semi-constant-time evolution for the indirect ^1^H dimension, with 4 scans per increment, a mixing-time of 0.4 s, a recycle delay of 1.0 s and *t*_1,max_ evolution times of 25 ms and 40 ms for the ^13^C and ^1^H indirect dimensions, respectively. The CCH-NOESY spectrum was recorded using a ^13^C-HMQC–NOESY–^13^C-HMQC pulse-sequence (Clore et al. 1991; Vuister et al. 1993) acquired in an (H)CCH fashion, with 8 scans per increment, a mixing-time of 0.4 s, a recycle delay of 1.0 s and *t*_1,max_ evolution times of 25 ms for both ^13^C indirect dimensions. Two 4D HCCH-NOESY spectra were recorded: the first on an LV[rac]-labelled sample (~ 550 µM in a 5-mm Shigemi tube) and the second on an IMLV[rac]-labelled sample (~ 550 µM in a 3-mm Shigemi tube). Both spectra were recorded using a ^13^C-HMQC–NOESY–^13^C-HMQC pulse-sequence that incorporated double semi-constant-time evolution for the indirect ^1^H dimension, together with non-uniform sampling (NUS) in all three indirect dimensions and diagonal-peak suppression. Poisson-gap sampling schedules were generated using the web-based ist@HMS schedule generator (Hyberts et al. 2013); all schedules were generated with the sinusoidal-weight parameter set to 2. Diagonal-peak suppression was implemented according to the approach of Wen et al. (2012), which requires two data-sets per final NOESY spectrum: a standard “all-peak” data-set, in which the time-domain data contains signals from both cross-peak and diagonal-peak correlations, and a “diagonal-peak” data-set, whose time-domain data contains only those signals from diagonal-peak correlations. The diagonal-peak data-set is then subtracted from the all-peak data-set prior to NUS reconstruction.

The first 4D HCCH-NOESY spectrum (LV[rac]-labelled sample) was recorded with identical acquisition parameters for the all-peak and diagonal-peak data-sets (8 scans per increment, a mixing time of 0.45 s and a recycle delay of 0.6 s; the *t*_1,max_ evolution times were 20 ms for both the indirect ^13^C dimensions and 19 ms for the indirect ^1^H dimension). The diagonal-peak-suppression was optimized by applying an empirical scaling factor of 1.45 to the diagonal-peak data-set prior to its subtraction from the all-peak data-set; the scaling-factor was estimated by reconstructing and processing both data-sets to yield the corresponding all-peak and cross-peak spectra and then inspecting the relative intensities of the diagonal peaks in the two spectra. The nominal NUS sparsity was 0.55% (~ 17% per indirect dimension), but the spectral widths for the three indirect dimensions were approximately twice the respective ranges of the observed signals (22 ppm in the two ^13^C dimensions and 4 ppm in the ^1^H dimension), translating into a Nyquist grid with approximately two-fold oversampling in all three dimensions and for which the number of hypercomplex points is a factor of 2^3^ greater than for the Nyquist grid that would correspond to a uniformly sampled data-set with the same *t*_1,max_ values in all dimensions but with spectral widths set to the ranges of the observed signals. A nominal NUS sparsity of 0.55% therefore translates to an effective NUS sparsity of 0.55 × 2^3^ = 4.4% (~ 35% per indirect dimension).

The second 4D HCCH-NOESY spectrum (IMLV[rac]-labelled sample) was recorded with a shorter mixing time for the diagonal-peak data-set (0.28 s), the length of which was optimized in an empirical fashion relative to that for the all-peak data-set (0.4 s) so that maximum suppression of diagonal peaks would be obtained without prior scaling of the diagonal-peak data-set. All other acquisition parameters were the same for both data-sets (8 scans per increment, a recycle delay of 0.6 s and *t*_1,max_ evolution times of 19 ms for both the indirect ^13^C dimensions and 20 ms for the indirect ^1^H dimension.) The nominal NUS sparsity was 0.21% (~ 13% per dimension), but the spectral widths were again set to twice the underlying signal ranges in the respective dimensions (39 ppm in the two ^13^C dimensions and 6 ppm in the ^1^H dimension), resulting in an effective NUS sparsity of 0.21 × 2^3^ = 1.7% (~ 26% per indirect dimension).

BN-BC intra-molecular PRE data were measured for paramagnetic tags attached at three positions within the BC domain: residue-numbers 102, 185 and 406 (corresponding to point-mutants S102C, A185C and S406C, respectively). The ^1^H,^13^C-HMQC spectra for A185C and S406C were recorded without the purge element and with *t*_1,max_ evolution times of 102 ms (^1^H) and 80 ms (^13^C). The spectra for S102C were recorded with the purge element and with *t*_1,max_ evolution times of 82 ms (^1^H) and 35 ms (^13^C).

The 3D HmCmCG/CB spectrum was recorded on the sample whose labelling scheme was described above (~ 300 µM in 5-mm Shigemi) using a pulse-sequence similar to that previously published by Sprangers and Kay (Sprangers and Kay 2007). Briefly, the magnetization-transfer-pathway comprises an outwards INEPT-based transfer (duration 1/(2*J*_HC_) ~ 4 ms) from Hm to Cm, followed by out-and-back COSY-like transfers (duration 1/(2*J*_CC_) ~ 14 ms) between Cm and CG (Leu) or CB (Val) sandwiching a central constant-time evolution period (duration 1/*J*_CC_ ~ 28 ms) for encoding the Leu CG and Val CB chemical shifts. The Cm chemical shifts are encoded in a constant-time fashion during the backwards CG/CB-to-Cm COSY transfer element before Cm magnetization is transferred back to Hm for detection in a methyl-TROSY fashion. The *t*_1,max_ evolution times for the indirect CG/CB and Cm dimensions were 25 ms and 12.5 ms, respectively.

### Data processing and analysis

Reconstruction of non-uniformly sampled time-domain data was achieved via iterative soft thresholding, as implemented in the software hmsIST provided by the group of Gerhard Wagner (Hyberts et al. 2012); parallelization was provided by the GNU command-line tool ‘parallel’ (Tange 2021). All uniformly sampled and reconstructed NUS time-domain data were processed with NMRPipe (Delaglio et al. 1995). Spectral visualization, analysis and assignment were done using CcpNmr Analysis v2.4 (Vranken et al. 2005). Extraction of quantitative peak-heights for the calculation of PRE intensity-ratios (see below) was done via peak-fitting using the program FuDA (Hansen 2007). Visualization and inspection of structures were done using PyMOL v.1.8–2.4 (DeLano, Schrödinger 2021).

#### Analysis of PRE data

PRE effects for the three point-mutants (S102C, A185C and S406C) were visualized both by calculating the ratios of the peak intensities (peak heights) in the paramagnetic spectra to those in the respective diamagnetic spectra (*I*_para_/*I*_dia_) and also by direct inspection of the overlaid spectra. Corresponding theoretical intensity-ratios were calculated from the structure of BC according to the following steps. First, models of the three mutants (including the attached spin-label) were built from the crystallographic structure of BC using Xplor-NIH (Schwieters et al. 2003), from which were extracted all methyl-proton–electron distances (using the average position of the three methyl protons and assuming the electron to be localised on the nitrogen atom of the spin-label nitroxide group). Next, the theoretical ^1^H transverse PRE, $\:{\varGamma\:}_{2}^{\text{H}}$, was calculated for each methyl-proton–electron distance, $\:{r}_{eN}$, according to the following simplified form of the Solomon-Bloembergen equation:

$$\:{\varGamma\:}_{2}^{\text{H}}=\frac{{\kappa\:}_{\text{H}}}{{r}_{eN}^{6}}{\mathcal{S}}^{2}{\tau\:}_{1}\left(4+\frac{3}{1+{\omega\:}_{0}^{2}{\tau\:}_{1}^{2}}\right)$$

where $\:{\mathcal{S}}^{2}$ and $\:{\tau\:}_{1}$ are the squared order parameter and effective correlation time for the electron–nucleus vector (set to 0.8 and 20 ns, respectively), $\:{\omega\:}_{0}$is the ^1^H Larmor frequency in rad·s^–1^ and $\:{\kappa\:}_{\text{H}}$ is given by:

$$\begin{aligned}{\kappa\:}_{\text{H}}&={\left({\gamma\:}_{\text{H}}{g}_{e}\beta\:\right)}^{2}\cdot{\left(\frac{{\mu\:}_{0}}{4\pi\:}\right)}^{2}\cdot\left(\frac{S\left(S+1\right)}{15}\right)\\&\quad=1.2311\times\:{10}^{16}{\dot A}^{6}{s}^{-2}\end{aligned}$$

in which physical constants are represented by their standard symbols and $$\:S$$ is the electronic spin quantum number ($$\:S=\raise.5ex\hbox{$\scriptstyle 1$}\kern-.1em/\kern-.15em\lower.25ex\hbox{$\scriptstyle 2$}$$ for the nitroxide radical used here). Finally, the theoretical intensity ratio was calculated according to:

$$\begin{aligned}&\frac{{I}_{\text{para}}}{{I}_{\text{dia}}}=exp\left(-{\varGamma\:}_{2}^{\text{H}}\varDelta\:\right)\\&\quad\frac{\left({R}_{2\text{,dia}}^{\text{HC-MQ}}+\pi\:\cdot{\text{LB}}_{\text{F1}}\right)\cdot\left({R}_{2\text{,dia}}^{\text{H-SQ}}+\pi\:\cdot{\text{LB}}_{\text{F2}}\right)}{\left({R}_{2\text{,dia}}^{\text{HC-MQ}}+\pi\:\cdot{\text{LB}}_{\text{F1}}+{\varGamma\:}_{2}^{\text{HC-MQ}}\right)\cdot\left({R}_{2\text{,dia}}^{\text{H-SQ}}+\pi\:\cdot{\text{LB}}_{\text{F2}}+{\varGamma\:}_{2}^{\text{H}}\right)}\end{aligned}$$

where $\:\varDelta\:$ is the total duration of the fixed delays in the ^1^H,^13^C-HMQC pulse-sequence (set to 7.7 ms), $\:{R}_{2\text{,dia}}^{\text{HC-MQ}}$ and $\:{R}_{2\text{,dia}}^{\text{H-SQ}}$ are the diamagnetic relaxation rates for ^1^H-^13^C multiple-quantum and ^1^H single-quantum coherences (both set to 40 s^–1^), $\:{\text{LB}}_{\text{F1}}$ and $\:{\text{LB}}_{\text{F2}}$ are exponential line-broadening factors in the indirect and direct dimensions (both set to 5 Hz), and $\:{\varGamma\:}_{2}^{\text{HC-MQ}}$ is the PRE for ^1^H-^13^C multiple-quantum coherence

$$\:{\varvec{\varGamma\:}}_{2}^{\text{HC-MQ}}\approx\:\left[1+{\left(\frac{{\varvec{\gamma\:}}_{\text{C}}}{{\varvec{\gamma\:}}_{\text{H}}}\right)}^{2}\right]\cdot{\varvec{\varGamma\:}}_{2}^{\text{H-SQ}}=1.063{\varvec{\varGamma\:}}_{2}^{\text{H-SQ}}$$

#### Chemical shift predictions

^1^H and ^13^C methyl (Ile-δ1, Leu-δ1/δ2, Val-γ1/γ2 and Met-ε) chemical shifts were predicted from the crystallographic structure of BC using two different chemical-shift prediction programs: SHIFTX2 (version 1.10) (Han et al. 2011) and CH3Shift (version 1.2.5) (Sahakyan et al. 2011). The two sets of predicted chemical shifts were combined into a single set; for nuclei whose chemical shifts were predicted by both programs (most cases), the entries in the combined set were calculated as the corresponding averages; otherwise, the single available predicted value was used. The estimated uncertainty of each averaged value was taken to be either the uncertainty generated by CH3Shift or the difference between the predicted values from CH3Shift and SHIFTX2 (whichever was larger). The uncertainties in entries for which only the CH3Shift predictions were available were taken simply as the respective CH3Shift uncertainties, while the uncertainties in entries for which only SHIFTX2 predictions were available were set to 0.2 ppm and 1.0 ppm for ^1^H and ^13^C shifts, respectively.

## Extent of assignments and data deposition

### Assignment strategy

Given the large size of BN-BC (~ 60 kDa), we did not attempt to assign the backbone resonances, and hence the overall strategy for assignment of the BC methyl groups was based on a “methyl-only” approach, exploiting the structural information provided by our determination of the crystallographic structure of the BC domain and the solution NMR structure of the BN domain (Karanth et al. 2024).

The domain architecture of BN-BC is of two well-structured domains, which are almost, but not entirely independent. At the outset of the project, comparative sequence analysis of the TomB gene product revealed that residues 91–533 constituted a canonical NRPS condensation domain, but no domain classification could be found for residues 1–90. The crystallographic structure of the BC domain was solved using crystals grown from the BN-BC construct, but no resolvable electron density was detected for residues 1–79. The crystallographic structure confirmed that BC consists of two sub-domains, designated as the N- and C- lobes, with comparable ILMV residues content. Subsequent NMR experiments revealed that the residues 4–77 do have a well-defined structure — adopting a PCP-like fold of a four-helix bundle— but that this domain (which we refer to as the BN domain) and the BC domain exhibit a significant degree of inter-domain flexibility within the context of the complete BN-BC construct. We subsequently solved the structure of the BN domain in isolation by solution NMR (Karanth et al. 2024), the process of which naturally included assignment of its methyl-groups. Minimal differences in chemical shifts of methyl groups were observed when comparing methyl spectra of BN with that of BN-BC, allowing an easy transfer of assignments of BN to di-domain BN-BC.

While this assignment note is principally concerned with the subsequent efforts to assign the methyl-groups of the BC domain, most of the experiments for this purpose were recorded on the BN-BC construct, since samples of this construct were more stable than those prepared from a BC-only construct, and other experiments had revealed that the BN domain — attached to BC in the context of BN-BC — plays a critical role in the overall function of the TomB module, being required for the specific recruitment of the peptidyl carrier domain of the upstream module.

**Fig. 2:**
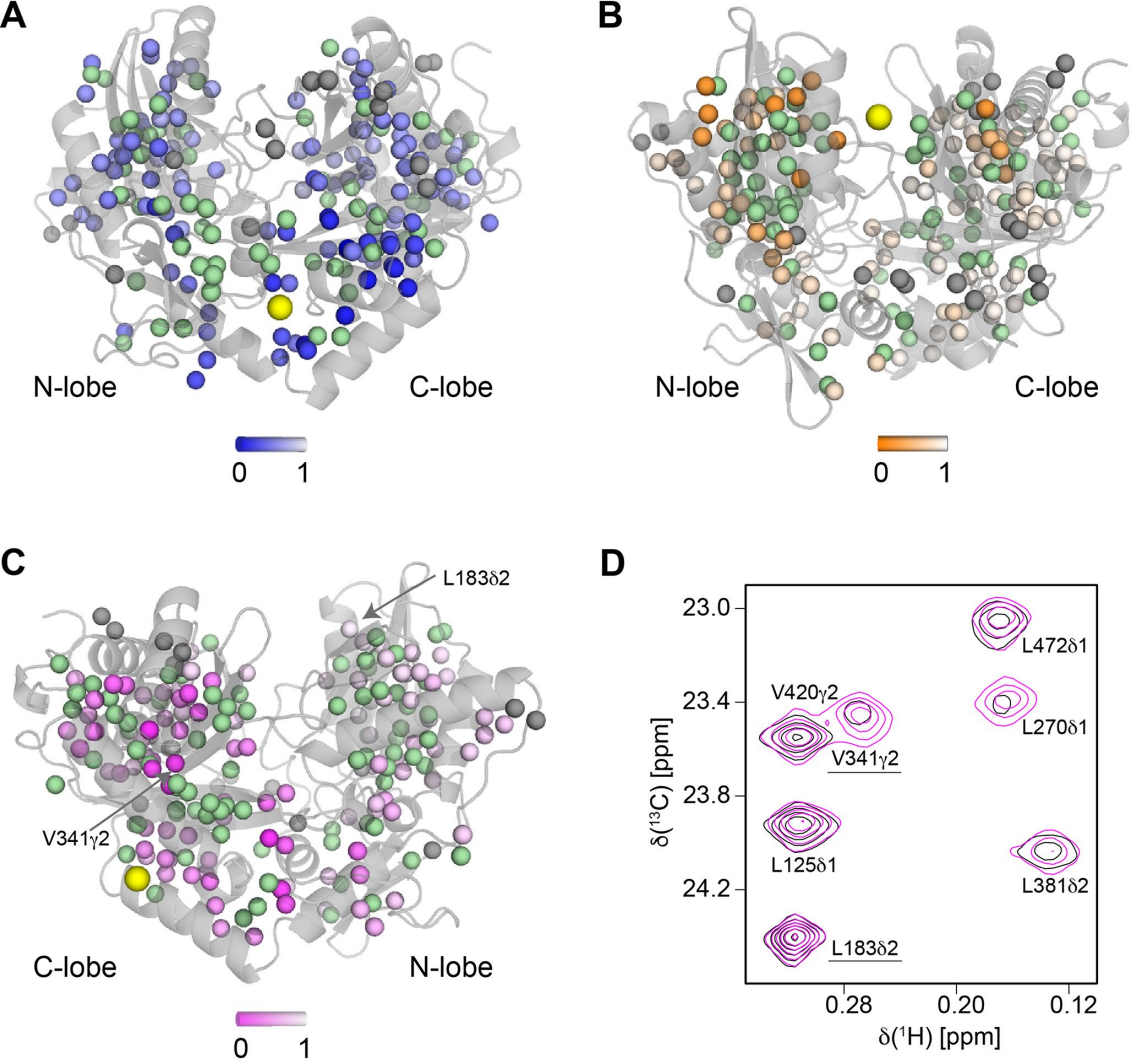
Extent of paramagnetic relaxation enhancement of BC methyl groups peaks caused by a paramagnetic tag attached to an individual, engineered cysteine residue in three single-point mutants. Information from peak intensity loss of methyl groups in spatial proximity to the tag was used to aid methyl groups’ assignment. **A–C.** Crystallographic structure of BC (light grey) with methyl groups represented by small spheres and the location of the PRE tag indicated by a yellow large sphere. Panels **A**–**C** are for the mutants S106C (**A**), A185C (**B**) and S410C (**C**). Spheres representing unassigned and assigned methyl groups are coloured in grey and green, respectively. Intensity loss is calculated as I_para_/I_dia_, where I_para_ and I_dia_ are the intensities of the methyl group peaks that could be accurately quantified in the paramagnetic and diamagnetic states, respectively. The values vary from 0 (severe) to 1 (no change) and are represented as a linear gradient from blue (**A**), orange (**B**), magenta (**C**) to white in each panel. **D.** Expansion of the overlay of the HMQC spectra of BC-S410C coupled to a spin-label in the paramagnetic (black) and diamagnetic (magenta) states. Two exemplary peaks are annotated and indicated in panel **C**: V341δ2, which shows substantial intensity loss, and L183δ2, which shows no intensity change

The ^13^C-HMQC methyl spectrum of ILV-labelled BN-BC revealed peaks with a wide range of intensities and linewidths, indicative of widespread dynamics on a range of timescales. In particular, there were more peaks than methyl groups, suggesting the presence of at least one dynamic process that is slow on the chemical-shift timescale. Initially, we tried to assign the methyl groups of BN-BC using two of the automatic methyl-group assignment programs available at the time (FLAMEnGO (Chao et al. 2014) and MAP-XSII (Xu and Matthews 2013), but we ultimately followed a completely manual approach. The broad strategy was to first provide a set of “anchor-point” assignments by inspection of the methyl-group spectra of BN-BC single-point mutants (mutated at one of a number of ad-hoc selected methyl-group-bearing residues) and then to extend the assignments from these anchor-points by analysing a suite of inter-methyl NOESY spectra in combination with the crystallographic structure of the BC domain. Mutations for initial assignments included residues with expected upfield-shifted proton signals because of large ring current effects, as judged from the structure of BC (Koradi et al. 1996). The approach was supplemented by incorporating information from a variety of other experiments and samples, including measurement of intra-domain paramagnetic relaxation enhancements (PREs) on three structurally distant single-point mutants (Fig. 2) and acquisition of an HmCmCG/CB-type experiment to pair-up the peaks of the two terminal methyl-groups of Leu and Val residues. In addition, we inspected the chemical shifts predicted from the structure by two chemical-shift-prediction programs (SHIFTX2 (Han et al. 2011) and CH3Shift (Sahakyan et al. 2011). The stereospecific assignments were obtained by analysis of the methyl spectra acquired on the MV[pro-S] and LV[pro-R] samples.

### Assignment coverage

**Fig. 3:**
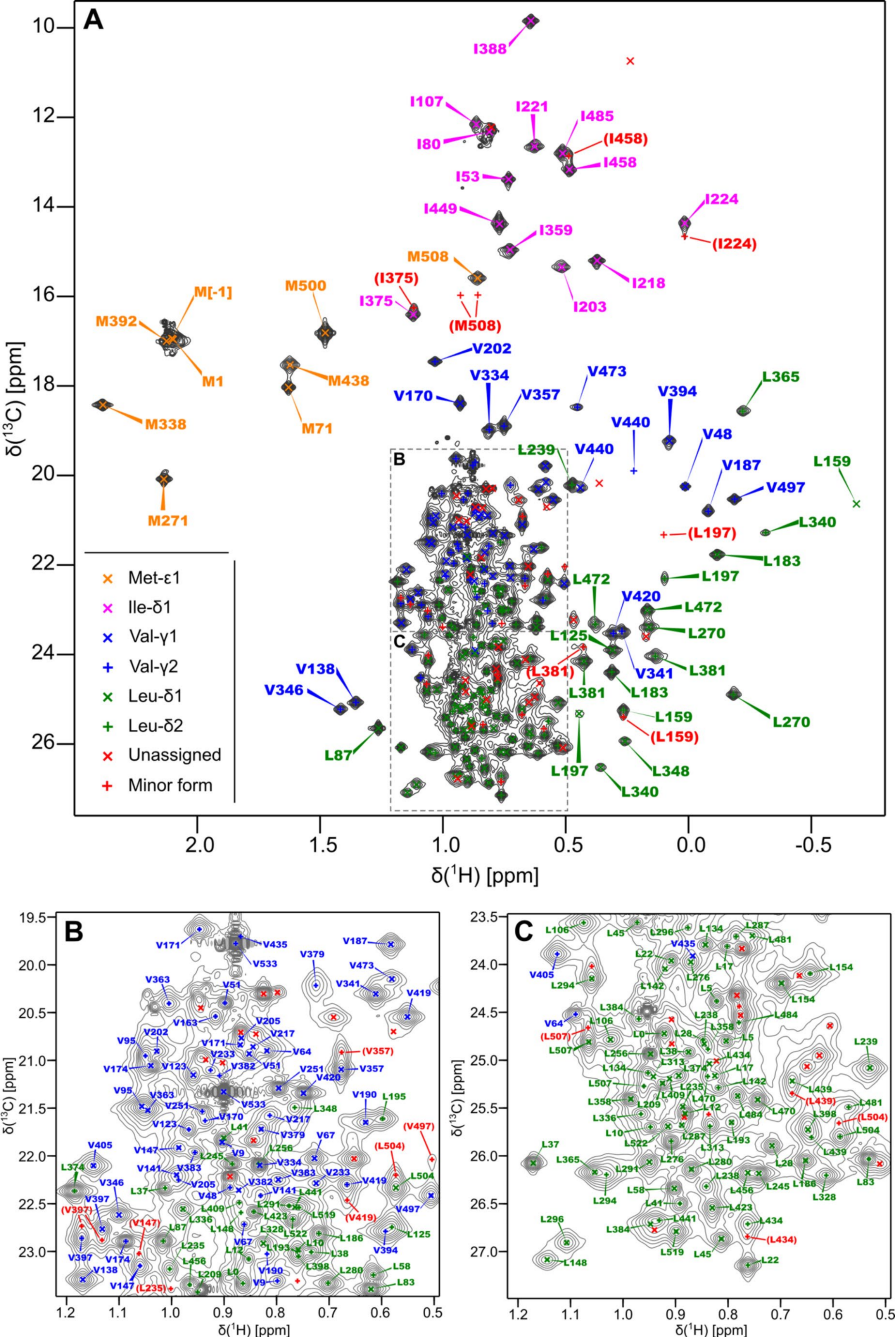
^1^H,^13^C-HMQC spectrum of IMLV[rac]-labelled BN-BC with available peak assignments (recorded on the 850-MHz spectrometer). **(A)** The complete spectrum, with peak-marks and annotations for the Met-ε (orange) and Ile-δ1 (magenta) peaks and the more dispersed Leu-δ1/δ2 (green) and Val-γ1/γ2 (blue) peaks. **(B) & C.** Expansions corresponding to the upper and lower halves, respectively, of the crowded central region, with peak-marks and annotations for the Leu-δ1/δ2 and Val-γ1/γ2 peaks therein. The Leu and Val pro-R peaks (Leu-δ1 & Val-γ1) are marked with cross (×) symbols while the corresponding pro-S peaks (Leu-δ2 & Val-γ2) are marked with plus (+) symbols. Peaks belonging to the minor form are marked with a red plus symbol and annotated with their proposed assignment (where available), while other unassigned peaks (which may belong to either the major or the minor form) are marked with a red cross symbol and remain unannotated

**Fig. 4:**
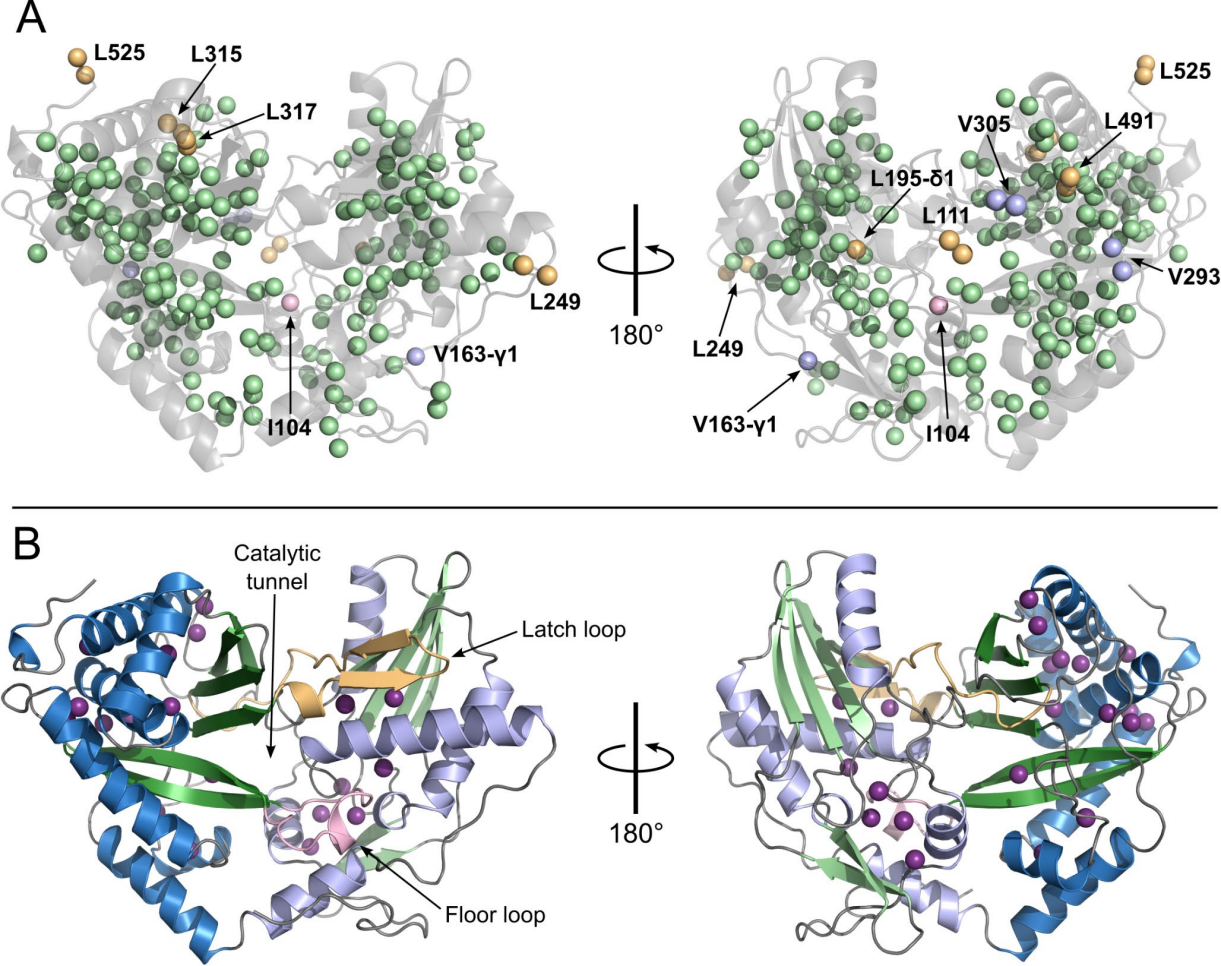
**(A)** Assignment status of the Ile, Met, Leu and Val (IMLV) methyl groups in the BC domain of BN-BC: shown are two views (related by a 180° rotation around the vertical axis) of the crystallographic structure of the BC domain with the IMLV methyl groups represented as coloured spheres; each sphere corresponds to a pseudo-atom located at the geometric centre of the three protons of the respective methyl group. The protein backbone is shown in light-grey semi-transparent ribbon representation. Spheres of assigned methyl groups are coloured pale-green; those of unassigned Ile, Leu and Val methyl-groups are coloured light-pink, light-orange and light-blue, respectively. **(B)** Structural features of the BC domain. The left and right views are the same two views of the BC domain as in panel A, with the backbone shown in solid ribbon representation. The N- and C-lobes correspond to the right and left lobes of the structure in the left view; helices of the N- and C-lobes are coloured light- and dark-blue, respectively, while the corresponding colour-scheme for β-strands is light- and dark-green (regions of non-canonical secondary structure are coloured grey). The floor- and latch-loop, forming cross-over elements between the two sub-domains, are coloured light-pink and light-orange, respectively. Also indicated is the position of the central catalytic tunnel. The donor and acceptor faces of BC domain — to which dock the upstream and downstream PCPs, respectively — correspond to the front and rear faces of the structure in the left view. Methyl groups for which a minor-form peak can be clearly distinguished and confidently assigned are represented as purple spheres. The BC structure depicted here is that of chain A of PDB: 8RZ6 (Karanth et al. 2024)

The final assignment is nearly complete. Overall, we have assigned 13 of the 14 Ile-δ1 methyl-groups, all eight Met-ε methyl-groups, 127 of the 140 Leu-δ1/δ2 methyl-groups and 75 of the 80 Val-γ1/γ2 methyl-groups; in total, 224 of the 243 ILVM methyl-groups (excepting Ile-γ2) have been assigned, corresponding to a coverage of 92%. The assigned ^1^H,^13^C-HMQC spectrum of IMLV[rac]-labelled BN-BC is shown in Fig. 3 and the assignment status of the BC methyl groups is depicted on the crystallographic structure of the BC domain in Fig. 4.

The remaining unassigned methyl-groups of the major-form are labelled on the structural views shown in Fig. 4A. For several of these methyl-groups, such as those of L249 and L525, and to a lesser extent L111 and L491, the lack of assignment is due principally to their structural isolation from other methyl-groups. For L529, there are no other methyl-groups within 12 Å, while the closest methyl-groups to L111, L249 and L491 lie at distances of 6.9 Å, 7.6 Å and 6.5 Å, respectively. Of the other unassigned methyl-groups, those of L315 and L317 proved to be particularly problematic. Despite belonging to a dense cluster of leucine residues, the candidate peaks for the methyl-groups of these two residues could not be confirmed with sufficient confidence to make formal assignments. These difficulties are partially due to the structural heterogeneity of the region — L315 and L317 are located within the region of the C-lobe that was revealed as one of the principal conformationally variable hotspots — and of L315 in particular. Comparison of the structures of the two chains of the crystal structure reveals significant differences in the positions of the methyl-groups of L315, with the χ_2_ dihedral angle corresponding to the *trans* rotameric state in chain A (χ_2_ = − 166.7°) and to the *gauche* + conformer in chain B (χ_2_ = + 49.1°). Protein harbouring the L315A mutation were unstable for NMR studies, highlighting the limitation of the point-mutation strategy for obtaining assignments.

In addition to peak-marks and annotations for the assigned peaks, Fig. 3 also includes peak-marks for peaks that have been designated as “minor form” and “unassigned”. As mentioned previously, the appearance of the BN-BC spectrum is characteristic of a protein exhibiting significant dynamics over a range of timescales. In particular, there is at least one dynamic process that is slow on the chemical-shift timescale, corresponding to conformational exchange between a major and a minor form. For some methyl-groups, it is possible to identify — with a reasonable degree of certainty — the peaks corresponding to both the major and minor form. Such identification is only straightforward when (a) both peaks appear in the less-crowded regions of the spectrum, (b) they arise from large enough conformational changes translating to large enough chemical shift differences between the two forms. These peaks are marked with a red plus symbol and annotated with the probable methyl-group in parentheses in Fig. 3; they are also shown on the structure of BC in Fig. 4B. It is likely that additional methyl-groups exhibit two forms, but that their minor-form peaks could not be identified or assigned because of spectral crowding. Finally, the peaks marked with a red cross symbol in Fig. 3 and designated as “unassigned” in the legend may belong to either the major or the minor form.

For a few methyl groups (e.g. M508), we identify not only the peak from the minor-form, but also an exchange-peak arising from exchange-mediated magnetization transfer during the final re-phasing delay of the HMQC pulse-sequence. In fact, several of the peaks annotated as minor-form peaks differ in their position relative to the corresponding major-form peak only with respect to their ^13^C chemical shift; the ^1^H chemical shift appears unchanged. Peaks of this sort may either be minor-form peaks of methyl-groups for which only the ^13^C chemical shift differs significantly between the two forms, or they may be exchange peaks whose ^13^C chemical shift corresponds to that of the minor-form but whose ^1^H chemical-shift is that of the major-form; heavy exchange-induced line-broadening effects in the ^1^H-dimension could cause minor-form peaks to become undetectable, while one of the two exchange peaks could remain detectable.

Despite the fact that more methyl-groups with major and minor forms can exist than those annotated in Fig. 3, the methyl-groups with assigned minor-form peaks are sufficient to pinpoint certain conformationally variable “hotspots” within BC that must undergo significant structural rearrangements in transitioning between the major- and minor-forms. These include the two pockets in the N-lobe that accommodate the latch-loop and floor-loop elements, which cross-over from the C- to the N-lobe, and a large region throughout and around the four-helix bundle (helices α7, α8, α9 & α11) that forms the outer part of the C-lobe (Fig. 4B).

To conclude, we have assigned the methyl-group resonances of the ~ 60-kDa dynamic BN-BC didomain using structural information of the individual domains and a variety of NMR-derived parameters, including NOE and PRE data. These assignments are essential to study the structure of the didomain in the context of the module it belongs to and to describe its interaction with the other domains. Furthermore, a thorough characterization of the dynamic conformational landscape of the prototypical condensation domain BC will provide mechanistic insights into the condensation reaction in NRPS machinery.

## Data Availability

The chemical shift assignments have been deposited in the Biological Magnetic Resonance Databank (BMRB) under accession number 52652.
